# Diagnostic performance of serum cystatin C and complement component 1q in lupus nephritis

**DOI:** 10.1186/s13075-019-2065-x

**Published:** 2019-12-04

**Authors:** Bei Xu, Ya-mei Zhang, Yu-wei Yang, Yun-shuang Liu, Jia-fu Feng

**Affiliations:** Department of Clinical Laboratory, Mianyang Central Hospital, Southwest Medical University, No.12 Changjiaxiang, Jingzhong Street, Mianyang, 621000 Sichuan China

**Keywords:** Lupus nephritis, Biomarker, Cystatin C, Complement component 1q

## Abstract

**Background:**

The information concerning non-invasive, easily obtainable, and accurate biomarkers for diagnosis of lupus nephritis (LN) is extremely limited. The aim of this study was to evaluate the diagnostic performance of cystatin C (CysC) and complement component 1q (C1q) for LN.

**Methods:**

A case-control study that included 905 patients with systemic lupus erythematosus (SLE) without LN (group SLE), 334 patients with active lupus nephritis (group LNA), 255 patients with inactive lupus nephritis (group LNI), and 497 healthy individuals (group HC) was performed in Mianyang Central Hospital from March 2017 to December 2018. The serum levels of CysC, C1q, urea (Urea), and creatinine (Creat) were measured, and 2 estimated glomerular filtration rates (eGFR_CysC_ and eGFR_Creat_) were calculated by equations which were based on serum CysC established by our group and the modification of diet in renal disease (MDRD), respectively. ANOVA analysis or Kruskal-Wallis test was used for comparing the differences among the groups, and receiver operating characteristic (ROC) curve was applied to identify the diagnostic efficiencies of individual or combined multiple indicators.

**Results:**

Significantly elevated CysC and decreased C1q were observed in the LNA and LNI groups, which was in contrast to their levels in the SLE and HC groups. CysC (AUC = 0.906) or eGFR_CysC_ (AUC = 0.907) assessed the highest diagnostic performance on LNA when detected individually, followed by C1q (AUC = 0.753). Joint utilization of C1q and CysC achieved very good performance (AUC = 0.933) which approximated to the best one observed in the combinations of C1q, Urea, CysC, eGFR_Creat_, and Creat (AUC = 0.975).

**Conclusion:**

The separately detected CysC (eGFR_CysC_) and C1q were superior to the conventional biomarkers Urea, Creat, and eGFR_Creat_ in the diagnosis of LNA. Moreover, although the combined detection of Urea, Creat, C1q, CysC, and eGFR_Creat_ had the greatest diagnostic performance, the joint utilization of CysC and C1q could be prioritized for rapid discrimination of LNA if the economic burden is taken into consideration.

## Introduction

Systemic lupus erythematosus (SLE) is a systemic autoimmune disease characterized by the involvement of diverse autoantibodies. The process of SLE pathogenesis can cause multiple tissue and organ damages [1]. When the kidney is involved, this condition can lead to lupus nephritis (LN) [2, 3]. LN was found to occur in 15–30% of the patients with lupus at the time of initial diagnosis and in 30–50% during disease progression [4]. Additionally, it severely negatively affects the SLE patients’ prognosis [5].

Accurate diagnosis and active treatment can preserve the kidney function of LN patients and delay the process of kidney fibrosis, thus postponing the occurrence and development of end-stage kidney disease [2]. Although a few lupus nephritis management guidelines have been published currently, they have been established in the USA and European countries, or are internationally based [6]. There is still little evidence to indicate their relative prediction and monitoring strategies in Asia. Additionally, serum or urine biomarkers such as serum creatinine and its clearance rate which are commonly used in the clinical evaluation of the kidney function in LN patients, as well as immune-related molecules, such as anti-double-stranded DNA antibody, anti-cardiolipin antibody, complement component C3b, and anti-C1q antibody, cannot display enough sensitivity and specificity to reflect the real-time renal immunopathological reactions [1, 7]. Since delayed diagnosis is related with a higher occurrence of end-stage kidney disease (ESKD) along with an unsatisfactory prognostication [7], rapid, accurate, and non-invasive diagnosis of kidney impairment is of substantial importance, especially in LN patients [8–11].

Cystatin C (CysC) has received much more attention than Creat as an alternative filtration marker with stronger and more linear risk relationships [12–14], which was also recommended by the KIDGO (2012) Chronic Kidney Disease Clinical Application Guideline. Several studies established that the addition of CysC measurements in the calculation of eGFR significantly improves the risk classification for death, cardiovascular disease, and ESKD [15–17]. Moreover, our group previously developed a glomerular filtration rate (GFR)-estimating equation based on CysC determination (eGFR_CysC_), which further confirmed it could achieve a much better diagnostic performance than that one based on Creat in Chinese patients with chronic kidney disease (CKD) [18]. Unfortunately, the value of eGFR_CysC_ in the diagnosis of kidney impairment in LN patients is still not validated.

Moreover, the complement system is one of the major effector mechanisms of the innate immune system that plays an important role in immune defense [19]. C1q is the initial complement component that activates the classical complement pathway and is critically involved in the clearance of immune complexes and apoptotic cell debris. Impaired clearance leads to exposure of C1 native antigen and development of anti-C1q antibody formation [20]. Hereditary deficiency of C1q is known to be a risk factor for the development of SLE [21]. The anti-C1q antibody has been well studied in SLE and has been proposed as a valuable biological marker in SLE with close association with renal involvement [22]. Conversely, only few published studies focused on serum C1q in SLE patients with LN [19, 23, 24]. Considering this, we designed this study to explore if serum C1q can contribute to the diagnosis of kidney impairment in LN patients as a novel disease biomarker.

Therefore, a case-control study that contained patients with SLE without renal involvement (group SLE), active lupus nephritis (group LNA), and inactive lupus nephritis (group LNI), as well as healthy individuals (group HC), was carried out in the present study. Their serum concentrations of urea, Creat, C1q, and CysC were measured, and Urea/Creat and eGFR (respectively based on CysC and Creat concentrations) were calculated. The main purpose of our study was to identify and establish combined biomarkers for detection from these traditional and new renal function indicators to achieve a more accurate and reliable diagnosis of LN.

## Methods

### Design

The present study is a case-control study, in which we analyzed the serum levels of the Urea, Creat (Urea/Creat), CysC (eGFR), and C1q of all subjects to evaluate their effectiveness in diagnosing LN. We included the patients who attended Mianyang Central Hospital (Sichuan, China) from March 2017 to December 2018 for treatment. They were categorized as with only SLE without renal involvement (group SLE), active lupus nephritis (group LNA), and inactive lupus nephritis (group LNI), all of whom fulfilled the inclusion criteria as described below. Otherwise, healthy controls (group HC) without systemic disease activity and with no history of renal disease that visited the hospital during the same period were recruited.

We analyzed the serum levels of the observed indicators of the subjects to evaluate their effectiveness in the diagnosis of LN. Receiver operating characteristic (ROC) curve was conducted to assess the performance of the new indicators in distinguishing LNA patients and healthy subjects in comparison with the frequently used ones such as Urea, Creat, and eGFR_Creat._

(1) Inclusion criteria: SLE was diagnosed according to the systemic lupus erythematosus guidelines by American College of Rheumatology Ad Hoc Committee [25], which was performed by serial or simultaneous presentation of at least 4 of the following 11 criteria: malar rash, discoid rash, photosensitivity, oral ulcers, non-erosive arthritis, serositis, renal dysfunction, neurological derangements (i.e., seizures or psychosis), hematologic disorder (i.e., anemia, leukopenia, thrombocytopenia), immunologic disorder (i.e., anti-DNA antibody, anti-Sm antibody, and false-positive VDRL testing), and presence of antinuclear antibodies. Kidney impairment was defined by a urinary albumin/creatinine ratio (UACR) ≥ 30 mg/g. LN was determined according to the American College of Rheumatology guidelines for screening, treatment, and management of lupus nephritis [26], which was defined as clinical and laboratory manifestations that meet the ACR criteria (persistent proteinuria > 0.5 g per day or greater than 3+ by dipstick, and/or cellular casts including red cell, hemoglobin, granular, tubular, or mixed). Systemic Lupus Erythematosus Disease Activity Index (SLEDAI) scores were calculated to evaluate the renal disease activity [27]. Patients with a SLEDAI score ≥ 4 were considered to have active lupus; otherwise, they were categorized as having inactive lupus.

(2) Exclusion criteria: patients with primary kidney disease, diabetes mellitus, cardiovascular dysfunction, respiratory dysfunction, ongoing infections, pregnancy, and other autoimmune diseases were excluded.

The study protocol was approved by the Medical Ethics Committee of Mianyang Central Hospital, and written informed consent was obtained from each patient before the study (S201400048, S2018085).

### Sample collection

Venous blood was collected into a BD Vacutainer® SST™ II ADVANCE tube (Becton Dickinson, USA) from all participants in the morning after overnight fasting. Then, the samples were mixed gently upside down eight times and left undisturbed for 30 min. The serum was separated by centrifugation at 3000 rpm for 15 min. All clinical laboratory tests were completed within 2 h after serum separation. Within 30 min after venous blood collection, the patient was instructed to provide approximately 10 mL of clean midstream urine for determination of urinary albumin and creatinine.

### Measurements of serum CysC, C1q, Urea, and Creat

Serum CysC, C1q, Urea, and Creat were measured using the fully automatic analyzer Labospect™ 008 (Hitachi, Japan). CysC and Urea kits were provided by Sichuan Maccura Biotechnology Co., Ltd. (Sichuan, China). C1q kits were obtained from Shanghai Beijia Chemical Reagent Co., Ltd. (Shanghai, China), and Creat kits were purchased from FUJIFILM Wako Pure Chemical Co., Ltd. (Osaka, Japan). Controls were respectively provided by Bio-Rad (Hercules, CA, USA); Sichuan Maccura Biotechnology Co., Ltd.; and Shanghai Beijia Chemical Reagent Co., Ltd. CysC was measured by turbidimetric immunoassay, and Urea and Creat were detected by the urease method and the sarcosine oxidase assay, respectively. Enzyme-linked immunosorbent assay (ELISA) is the frequently used method for C1q measurement. However, it has poor repeatability for quantitative detection. In this work, serum C1q levels were directly determined by automated immunoturbidimetric analysis. Compared to ELISA assays, its detection speed is much higher, and less interference and low cross-contamination between samples are observed.

### Calculation of estimated GFR

The eGFR formula based on serum CysC in Chinese patients established by our group was used to calculate the eGFR_CysC_ [18]. Otherwise, the modification of diet in renal disease (MDRD) equation developed by a Chinese group was also used to calculate eGFR_Creat_ [28]. The following formulas were used for the calculations:

$$ {\mathrm{eGFR}}_{\mathrm{CysC}}=78.64\times {\mathrm{CysC}}^{-0.964} $$

$$ {\mathrm{eGFR}}_{\mathrm{Cr}\mathrm{eat}}=175\times {\mathrm{Cr}}^{-1.234}\times {\mathrm{Age}}^{-0.179}\ \left(\mathrm{if}\ \mathrm{male}\right) $$

$$ {\mathrm{eGFR}}_{\mathrm{Cr}\mathrm{eat}}=175\times {\mathrm{Cr}}^{-1.234}\times {\mathrm{Age}}^{-0.179}\times 0.79\ \left(\mathrm{if}\ \mathrm{female}\right) $$

### Statistical analysis

All data are expressed as mean ± standard deviation (SD) or as the median (M) and quartile (P25, P75). The normal distribution was tested with the Kolmogorov-Smirnov method and Q-Q chart. Quantitative data with normal distribution were compared by one-way analysis of variance (ANOVA), whereas those with non-normal distribution were subjected to the Kruskal-Wallis non-parametric test. The non-parametric Spearman rank correlation coefficient was used for the statistical analysis of the data. The diagnostic efficiencies of individual or multiple indicators were analyzed with receiver operating characteristic (ROC) curve in which the area under the curve (AUC) was tested with DeLong non-parametric test. According to the maximum Yoden index (YI = sensitivity + specificity − 1), the cutoff value of each observed index was selected. Statistical analysis was performed with SPSS 19.0 (SPSS, Inc., Somers, NY, USA) and MedCalc11.5 (MedCalc Software, Mariakerke, Belgium). A value of *P* < 0.05 was considered statistically significant.

## Results

A total number of 1494 patients were recruited, including 125 men and 1369 women with a mean age of 39.2 ± 12.8 years (range 13–82 years). These patients were further divided into 3 groups: 905 SLE patients without renal involvement (group SLE), 334 patients with active lupus nephritis (group LNA), and 255 patients with inactive lupus nephritis (group LNI). Additionally, 497 healthy individuals, including 260 men and 237 women, with a mean age of 46.2 ± 13.8 years (range 11–85 years) served as healthy controls (group HC). Statistical analysis revealed significant differences in gender (*χ*^2^ = 461.777, *P* < 0.001) and age (*t* = 9.962, *P* < 0.001) between the patient group and the control group. Urinary albumin and creatinine measurements were employed to calculate UACR to further divide the patients into subgroups. These results had been presented in Table 1.

**Table 1:** Demographic characteristics and laboratory measurements of the studied subjects

	HC (*n* = 497)	SLE (*n* = 905)	LNA (*n* = 334)	LNI (*n* = 255)	*F*∕*χ*^2^	*P*
Demographic characteristics						
Male, *n* (%)	260 (52)	57 (6)	36 (12)	32 (13)		
Female, *n* (%)	237 (48)	848 (94)	298 (88)	223 (87)		
Age (years)	46.2 (11~85)	39.2 (13~82)	38.44 (13~76)	40.3 (13~80)		
Laboratory measurements						
UACR (mg/g), M (P25, P75)	8.15 (4.52,10.7)	9.31 (4.43, 12.07)	1197.00 (557.49,2212.39)	131.73 (57.01, 220.48)		
Urinary protein (g/24 h), M (P25, P75)	0.08 (0.04, 0.11)	0.14 (0.09, 0.20)	2.47 (0.95, 3.43)	0.18 (0.08, 0.27)		
Creat (μmol/L)	65.2 ± 12.97	55.1 ± 11.44^a^	62.2 (50.7, 87.4)^b^	61.2 ± 23.25^abc^	217.834	0.000
Urea (mmol/L)	5.11 ± 1.17	4.93 ± 1.58^a^	6.15 (4.58,8.88)^ab^	5.74 ± 2.17^bc^	131.728	0.000
Urea/Creat	0.08 ± 0.02	0.09 ± 0.03^a^	0.10 ± 0.04^ab^	0.10 ± 0.03^ab^	29.657	0.000
CysC (mg/L)	0.80 ± 0.13	0.96 ± 0.23^a^	1.22 (1.01, 1.75)^ab^	1.09 ± 0.41^abc^	550.624	0.000
eGFR_CysC_ [mL/(min·1.73 m^2^)]	100.3 ± 16.33	85.5 ± 17.12^a^	62.9 ± 23.92^ab^	78.5 ± 18.68^abc^	283.328	0.000
eGFR_Creat_ [mL/(min·1.73 m^2^)]	121.0 ± 24.37	139.9 ± 36.67^a^	112.7 ± 52.76^ab^	131.6 ± 40.82^abc^	52.886	0.000
C1q (mg/L)	182.29 ± 28.91	170.58 ± 35.52^a^	153.24 ± 39.57^ab^	170.80 ± 36.16^ac^	46.330	0.000

CysC and C1q were measured by turbidimetric immunoassay, and Urea and Creat were detected by the urease method and sarcosine oxidase assay, respectively. ANOVA analysis or Kruskal-Wallis test was used for comparing the differences among the multiple groups. *F*∕*χ*^2^ and *P* represented the statistical results of ANOVA analysis or Kruskal-Wallis test among all the study groups

*Urea* urea, *Creat* creatinine, *CysC* cystatin C, *C1q* complement component 1q, *UACR* urinary albumin to creatinine ratio

^a^Compared with the HC group, *P* < 0.05

^b^Compared with the SLE group, *P* < 0.05

^c^Compared with the LNA group, *P* < 0.05

### Serum levels of CysC, C1q, Urea, and Creat

The normal distribution test by the Kolmogorov-Smirnov method and Q-Q chart showed that all parameters had a normal or approximately normal distribution, except for CysC, Urea, and Creat in the LNA group. Thus, the Kruskal-Wallis non-parametric test was applied for statistical comparisons between the multiple groups for these parameters, whereas ANOVA was performed for the others. The serum levels of CysC, C1q, Urea, and Creat determined by the laboratory examinations are listed in Table 1; statistically significant differences were present among the study groups (all *P* < 0.001). The multiple comparisons (Table 2) showed that significant differences were available in CysC, eGFR_CysC_, and C1q between the healthy subjects and those diagnosed with SLE, LNI, and LNA (all *P* < 0.001), indicating that the elevated CysC and the decreased eGFR_CysC_ and C1q levels might serve as meaningful biomarkers for both SLE and LN diagnoses. To further distinguish the different disease status, multiple comparisons in groups SLE, LNI, and LNA were conducted; significant differences (all *P* < 0.001) of CysC, eGFR_CysC_, and C1q among these groups were found, except for the similar mean concentrations of C1q observed between SLE and LNI groups (*P =* 0.928). These results suggest that using only C1q is not sufficiently precise to differentiate subjects with SLE and LNI, but might be potentially used to reveal renal disease in patients with active and inactive LN. For the other traditional parameters, similar levels of Urea in the HC and LNI groups (*P* = 0.054) were established, whereas significant differences among the other groups were observed in these indicators (all *P* < 0.05). The differences in Creat among the groups were all statistically significant (all *P* < 0.05), except for that between the HC and LNA groups (*P* = 0.645). The ratios of Urea/Creat, had similar values only in the LNA and LNI groups (*P* = 0.917), whereas significant differences in eGFR_Creat_ were available among all groups (all *P <* 0.05).

**Table 2:** Multiple comparisons between the study groups

Items	Comparison pairs						
		HC to SLE	HC to LNA	HC to LNI	SLE to LNA	SLE to LNI	LNA to LNI
Urea	*z*	3.117	− 7.605	− 2.612	− 11.12	− 5.293	4.051
	*P*	0.011	0.000	0.054	0.000	0.000	0.000
Creat	*z*	13.378	1.610	6.66	− 9.887	− 3.299	4.799
	*P*	0.000	0.645	0.000	0.000	0.006	0.000
Urea/Creat	LSD-t	− 6.412	− 8.119	− 7.346	− 3.381	− 2.932	0.104
	*P*	0.000	0.000	0.000	0.001	0.003	0.917
CysC	*z*	− 13.005	− 22.801	− 13.561	− 13.857	− 4.493	6.838
	*P*	0.000	0.000	0.000	0.000	0.000	0.000
e-GFR_CysC_	LSD-t	14.340	28.624	15.292	19.128	5.322	− 10.189
	*P*	0.000	0.000	0.000	0.000	0.000	0.000
eGFR_Creat_	*LSD*-t	− 8.933	3.053	− 3.629	11.163	3.093	− 5.959
	*P*	0.000	0.002	0.000	0.000	0.002	0.000
C1q	LSD-t	6.024	11.789	4.283	7.775	− 0.091	− 6.063
	*P*	0.000	0.000	0.000	0.000	0.928	0.000

ANOVA analysis or Kruskal-Wallis test was used for comparing the differences among the multiple groups. *z*/LSD-t and *P* were the statistical results for the comparison between the two groups. LSD-t was the statistics of ANOVA analysis to find the difference between the observed indicators of the normal distribution, and *z* was the statistics of Kruskal-Wallis non-parametric test to find the difference between the two observed indicators of the non-normal distribution

*Urea* urea, *Creat* creatinine, *CysC*, *cystatin C*, *C1q* complement component 1q

### Analysis of the correlation between the observed indicators

As can be seen in Table 3, Spearman’s correlation analysis revealed significant correlations between the CysC levels and Urea, Creat, eGFR_CysC_, and eGFR_Creat_ in the HC, SLE, LNA, and LNI groups. The CysC levels were positively correlated with the values of Urea and Creat, whereas they were negatively correlated with eGFR_CysC_ and eGFR_Creat_. In the HC group, negative correlations were also observed between CysC and Urea/Creat (*P* < 0.001). By contrast, no significant correlations between C1q and other parameters were found among the four groups.

**Table 3:** Spearman’s correlation analysis of various parameters

			Urea	Creat	Urea/Creat	CysC	eGFR_CysC_	eGFR_Creat_	C1q
HC group	CysC	*r*	0.225	0.561	− 0.223	1	− 0.977	− 0.538	0.092
		*P*	0.000	0.000	0.000	–	0.000	0.000	0.040
	C1q	*r*	0.028	− 0.056	0.086	0.092	− 0.089	0.055	1
		*P*	0.534	0.217	0.055	0.040	0.048	0.218	–
SLE group	CysC	*r*	0.289	0.548	− 0.042	1	− 0.945	− 0.478	− 0.020
		*P*	0.000	0.000	0.204	–	0.000	0.000	0.556
	C1q	*r*	− 0.012	0.039	− 0.032	− 0.020	− 0.016	− 0.068	1
		*P*	0.721	0.242	0.342	0.556	0.632	0.041	–
LNA group	CysC	*r*	0.656	0.716	− 0.099	1.000	− 1.000	− 0.728	− 0.039
		*P*	0.000	0.000	0.071	–	0.000	0.000	0.473
	C1q	*r*	0.000	− 0.029	0.012	− 0.039	0.039	− 0.011	1.000
		*P*	0.996	0.603	0.832	0.473	0.474	0.838	–
LNI group	CysC	*r*	0.661	0.819	− 0.037	1	− 0.883	− 0.684	0.103
		*P*	0.000	0.000	0.552	–	0.000	0.000	0.101
	C1q	*r*	0.068	0.039	0.006	0.103	− 0.076	− 0.109	1
		*P*	0.280	0.537	0.924	0.101	0.228	0.082	–

Spearman’s correlation analysis was applied to identify the associations between CysC or C1q and other parameters

*r* correlation coefficient, *CysC* cystatin C, *C1q* complement component 1q

### Diagnostic efficiencies of individual or multiple biomarkers for kidney impairment in patients with LN

**Fig. 1:**
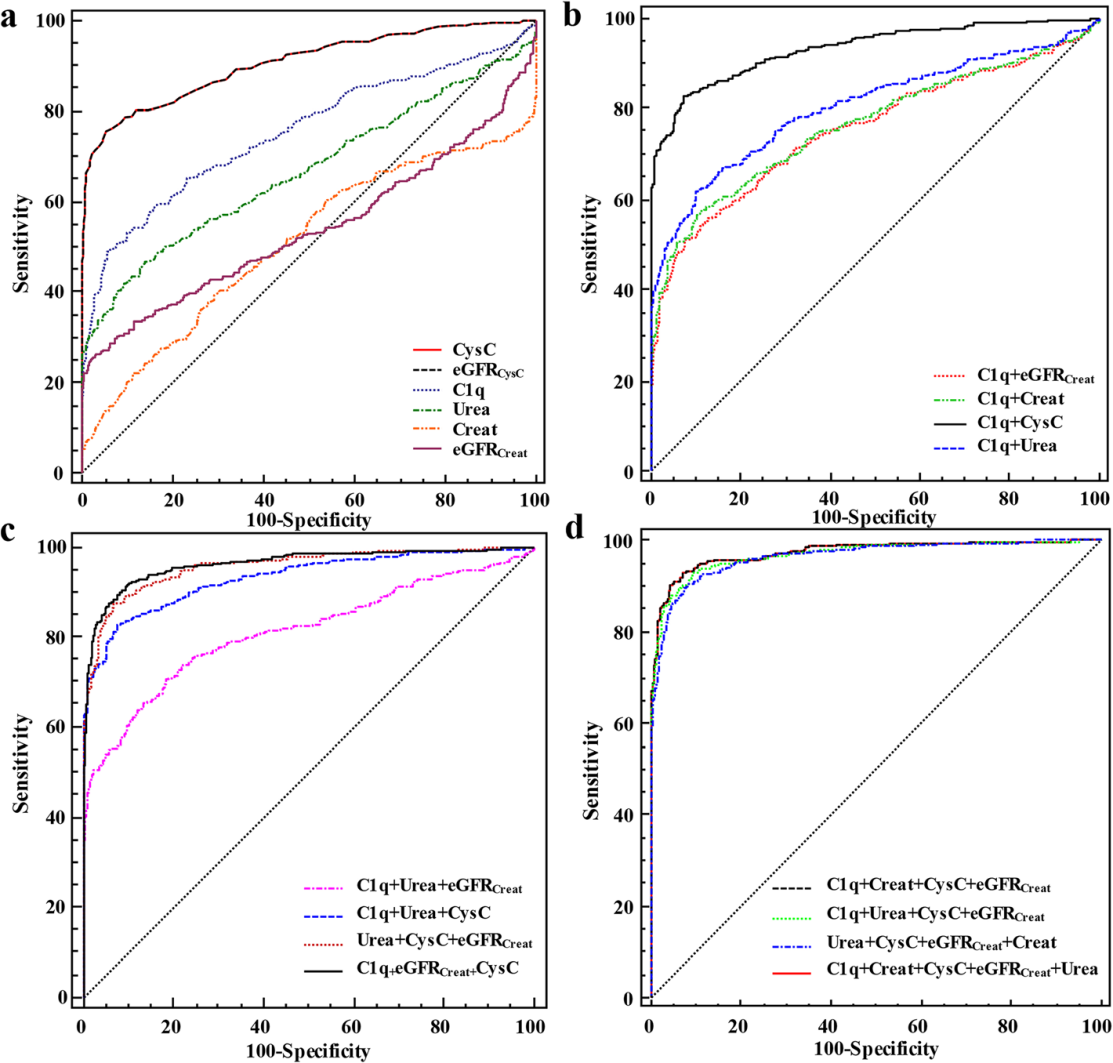
ROC analysis of single or combined biomarkers of LNA. **a** Individual biomarker’s detection. **b** Combined detection of two biomarkers. **c** Combined detection of three biomarkers. **d** Combined detection of multiple biomarkers

ROC analysis was employed to analyze the diagnostic efficiencies of the single and combined detection potential of CysC, eGFR_CysC_, C1q, Creat, eGFR_Creat_, Urea, and Urea/Creat for kidney impairment in LNA patients (Fig. 1). The value of each observed index corresponding to the maximum YI was selected as the cutoff value of the observed index. Creat (76.0%) had the highest individual detection sensitivity, whereas eGFR_Creat_ (25.1%) had the lowest sensitivity. Additionally, eGFR_Creat_ had the highest specificity (98.0%), whereas the lowest was observed in Creat (30.0%) (Table 4). Further analysis by the DeLong non-parametric test was performed for the AUC of the different parameters. AUC of CysC (AUC = 0.906) or eGFR_CysC_ (AUC = 0.907) was significantly higher than that of C1q (AUC = 0.753), Urea (AUC = 0.668), Urea/Creat (AUC = 0.639), eGFR_Creat_ (AUC = 0.539), and Creat (AUC = 0.508) (all *P* = 0.000). The finding revealed that CysC or eGFR_CysC_ had the best diagnostic performance for LN, followed by C1q when separately detected as a single indicator.

**Table 4:** Diagnostic efficiency of the individual biomarker of LNA

Items	AUC (95% CI)	*Z*/*P*^a^	Cutoff *V*	Se (%)	Sp (%)	YI	*Z*/*P*^b^
Creat	0.508 (0.473~0.542)	0.346/0.729	90.1	76.0	30.0	0.209	13.816/0.000
eGFR_Creat_	0.539 (0.504~0.573)	1.731/0.084	79.65	25.1	98.0	0.231	18.447/0.000
Urea/Creat	0.639 (0.605~0.672)	6.836/0.000	0.09	55.4	68.4	0.238	11.141/0.000
Urea	0.668 (0.634~0.700)	8.144/0.000	6.40	46.1	86.5	0.326	11.976/0.000
C1q	0.753 (0.723~0.782)	13.753/0.000	144	49.1	94.4	0.435	7.015/0.000
CysC	0.906 (0.884~0.925)	35.891/0.000	1.00	75.7	94.6	0.703	–
eGFR_CysC_	0.907 (0.885~0.926)	36.100/0.000	77.89	75.7	94.6	0.703	–

Receiver operating characteristic (ROC) curve was applied to identify the diagnostic efficiencies of individual indicator. *Z* was the statistics of DeLong non-parametric test to evaluate the statistical difference between the two AUCs

*Creat* creatinine, *Urea* urea, *C1q* complement component 1q, *CysC* cystatin C

^a^*Z* and *P* values were the AUC-based statistics of each item

^b^*Z* and *P* values were the AUC-based statistics of each item in comparison with eGFR_CysC_

In terms of combined detection, the diagnostic performance of LNA was the highest in its combinations with C1q, Urea, CysC, eGFR_Creat_, and Creat (AUC = 0.975), whereas those of C1q and eGFR_Creat_ were the lowest (AUC = 0.756) (Table 5). Statistically significant differences regarding diagnostic efficiency were found between the best combination and the other joint detections (all *P* < 0.05), except the combined utilization of C1q, CysC, eGFR_Creat_, and Creat (AUC = 0.974, *P* = 0.310). The joint detection of C1q and CysC achieved the highest diagnostic accuracy (AUC = 0.933) with high sensitivity (82.9%) and specificity (92.6%), which was slightly lower than those of the best three or the combined biomarkers (Table 5). These findings implied that the combined utilization of C1q and CysC could be a potential biomarker for diagnosis in LNA if the economic burden is taken into consideration.

**Table 5:** Diagnostic efficiencies of combined biomarkers of LNA

Items	AUC (95%CI)	*Z*/*P*^a^	Se (%)	Sp (%)	YI	*Z*/*P*^b^
C1q + eGFR_Creat_	0.756 (0.725~0.785)	13.926/0.000	51.2	92.2	0.434	12.196/0.000
C1q + Creat	0.765 (0.735~0.794)	4.587/0.000	57.5	88.9	0.464	11.864/0.000
C1q + Urea	0.805 (0.776~0.831)	18.108/0.000	62.0	89.9	0.519	10.627/0.000
C1q + CysC	0.933 (0.914~0.949)	46.787/0.000	82.9	92.6	0.755	6.172/0.000
C1q + Urea + eGFR_Creat_	0.813 (0.785~0.839)	8.969/0.000	70.7	81.7	0.524	10.309/0.000
C1q + Urea + CysC	0.934 (0.915~0.950)	46.787/0.000	82.9	92.6	0.755	6.182/0.000
Urea + CysC + eGFR_Creat_	0.959 (0.943~0.971)	67.443/0.000	87.7	93.2	0.809	4.178/0.000
C1q + CysC + eGFR_Creat_	0.969 (0.955~0.980)	79.306/0.000	92.8	90.7	0.836	3.802/0.001
Urea + CysC + eGFR_Creat_ + Creat	0.964 (0.949~0.976)	73.979/0.000	90.4	91.8	0.822	3.053/0.002
C1q + Urea + CysC + eGFR_Creat_	0.969 (0.955~0.980)	79.306/0.000	92.8	90.7	0.836	3.897/0.001
C1q + CysC + eGFR_Creat_ + Creat	0.974 (0.961~0.984)	88.822/0.000	91.0	95.0	0.860	1.016/0.310
C1q + Urea + CysC + eGFR_Creat_ + Creat	0.975 (0.962~0.984)	88.976/0.000	92.5	94.4	0.869	―

Receiver operating characteristic (ROC) curve was applied to identify the diagnostic efficiencies of combined multiple indicators

*Z* was the statistics of DeLong non-parametric test to evaluate the statistical difference between the two AUCs

*Creat* creatinine, *Urea* urea, *C1q* complement component 1q, *CysC* cystatin C

^a^*Z* and *P* values were the AUC-based statistics of each item

^b^*Z* and *P* values were the AUC-based statistics of each item in comparison with the joint utilization of C1q, Urea, CysC, eGFR_Creat_, and Creat

## Discussion

The identification of new clinical and laboratory biomarkers in the Asian population is crucial for the accurate determination of the renal involvement in LN diagnosis and supervision.

In this work, we evaluated for the first time the accuracy and suitability of the application of CysC, eGFR_CysC_, and C1q as biomarkers in the diagnosis of renal impairment in LNA patients from the Chinese population in clinical settings in China.

Our results showed unsatisfactory diagnostic performances of separately detected Creat, Urea, their calculated eGFR_Creat_, and Urea/Creat in LNA patients (AUC 0.508–0.668). Hence, we suggest that kidney impairment might precede the changes in Creat and Urea in LNA patients, and the traditional parameters would fail to diagnose early-stage kidney impairment in these populations. Some reasons which might affect the values of Creat were present as follows: (I) many physical factors (such as age, gender, and muscle mass) influence Creat levels much and make the individual difference large; (II) serum Creat is a later biomarker of kidney injury, for that, the rise in serum Creat is only observed when kidney function is decreased by 50% [29]. These various intra-variabilities for Creat might contribute to the different levels between healthy controls and the patient cases in our study. Additionally, the effects of Creat may also be the main reason for the difference between the results of eGFR_Creat_ (calculated based on Creat) in healthy controls as compared with the cases. Importantly, since eGFR_Creat_ is influenced by the various factors mentioned above, eGFR_CysC_ (calculated based on CysC) would be more effective than eGFR_Creat_ to represent the reduced GFR status in the disease groups in our study.

In contrast to the serum Creat concentrations, CysC was not affected by intra-variabilities such as gender and muscle mass, providing a more accurate measure of renal function [30, 31]. Evidence exists of the significant association of higher serum levels of CysC with increased microalbuminuria and lower GFR, which could reflect renal damage and impaired renal function [30]. We obtained similar results with significantly higher CysC but lower eGFR_CysC_ values in SLE, LNA, and LNI groups than those in the healthy controls, which indicates the existence of a close association between CysC/eGFR_CysC_ and SLE, especially in renal function impairment (LN). Additionally, the diagnostic efficiency of Creat in LNA patients was consistently lower than that of CysC, which further implied that Creat is inferior to CysC in the diagnosis of kidney impairment. eGFR is the clinical standard for the assessment of kidney function [32, 33]. Although the two indicators eGFR_CysC_ and eGFR_Creat_ were both applied in the Chinese group, our findings showed that eGFR_CysC_ had a higher performance in the diagnosis of kidney impairment in LNA. Moreover, the evidence is available that the measurement of Creat to determine the eGFR is limited by the influence of age and sex, as well as the variability in the clinical methodology for Creat determination [34, 35]. As known, the enzyme method is widely used in the majority of laboratories. Thus, the eGFR_CysC_ formula, which was previously established by our group, was confirmed to be more suitable and accurate for the diagnosis of LNA in Chinese SLE patients, revealing that careful and complete consideration is required to choose the most appropriate eGFR equation for the specific clinical application to avoid misdiagnosis and treatment errors. It also should be noted that eGFR is not a measured parameter and is calculated based on the values of Creat and/or CysC as well as those of other parameters. Thus, the results may be unavoidably affected by other factors. Hence, a specific threshold should be established in each individual lab, which can contribute to the accurate evaluation of kidney function.

The role and clinical relevance of the serum C1q levels in the renal involvement in LN patients still need to be elucidated. Our findings showed that the levels of C1q were significantly lower in the SLE, LNA, and LNI groups than in the normal controls. The C1q concentrations in the LNA were lower group than those in the LNI and SLE groups. These results are consistent with the ones in previous studies [24, 36], implying that the deficiency of C1q is a risk factor for the development of SLE, and the levels of C1q are closely associated with the development of renal disease in LN. Otherwise, no statistically significant correlations between C1q and other parameters were found among the HC, SLE, LNA, and LNI groups. Therefore, the serum complement C1q might serve as an independent risk factor in SLE and LN. To the best of our knowledge, we for the first time further evaluated the role of serum C1q in the diagnosis of kidney impairment in Chinese LNA patients. ROC curve analysis showed that C1q was superior to the widely used parameters Creat/eGFR_Creat_ in the diagnosis of kidney impairment in SLE patients with LNA. Therefore, we discovered that the individually detected serum C1q levels can serve as a novel and sensitive non-invasive biomarker for renal damage in LNA.

Because of the heterogeneity of LN, however, the simple use of just one parameter may hardly achieve satisfactory diagnostic performance [3]. The absence of a correlation between C1q and the other biomarkers suggested that the parameters investigated in this study might be related to different aspects of LN. Hence, the combined detection of C1q and other serological biomarkers can significantly improve the diagnostic accuracy. As expected, the diagnostic efficiencies of the combined detection of C1q and other markers were higher than that of the determination of these parameters alone. The diagnostic accuracy of LNA was the highest in the combinations of the five parameters tested, including C1q, Urea, CysC, eGFR_Creat_, and Creat. Interestingly, the combined detection of C1q, CysC, and eGFR_Creat_ could also achieve high diagnostic performance. But when taken the economic burden into consideration, the joint biomarkers of C1q and CysC could be a priority; for that, its diagnostic efficiency was only slightly lower than that of the highest one. In agreement with previous reports [37], our findings confirmed that the determination of a single index cannot accurately be used for disease diagnosis, and the joint detection of a range of indicators is more precise possibly due to their complementary roles.

Overall, compared to the renal biopsy which cannot be routinely conducted serially and unable to reflect the global renal pathological status due to the obtained small-size specimens, and 24 h urine protein quantification which needs to collect the patient’s urine with a long period for 24 h and cumbersome operation, our investigated biomarkers are more convenient, acceptable, and non-invasive with excellent sensitivity and specificity. They would offer an alternative to the rapid discrimination of renal damage in LN.

Although our study revealed that CysC/eGFR_CysC_ and C1q could be promising biomarkers for the identification of LNA in SLE patients, some limitations have to be acknowledged. First, the scope of our investigation was limited by its small sample size. Second, the data obtained were collected only at a single institution, and no follow-up data were available. Third, LN was determined mainly by laboratory parameters and clinical manifestations rather than by pathogenic analysis of renal biopsies. Moreover, the sex difference had not been taken into consideration, although the recognition and identification of sex-specific biological processes would have led to a better understanding of the parameters’ alterations in the diagnostic performance in LN patients. Therefore, in the future, we plan to conduct a multi-center study with larger sample sizes and rigorous inclusion of incident patients to further investigate the diagnostic values of CysC/eGFR_CysC_ and C1q in LN patients in China. Moreover, the validity of these observed indicators for the LN diagnosis in comparison with the gold standard tests such as kidney biopsy will be also needed.

## Conclusions

The significance of the values of CysC/eGFR_CysC_ and C1q in the diagnosis of renal impairment in LN patients was validated for the first time in China. Moreover, our finding confirmed that the deficiency of serum C1q is a risk factor for the development of SLE, and the levels of C1q are closely associated with the activity of LN. Therefore, C1q could serve as a novel and sensitive non-invasive single biomarker for renal damage. Additionally, apart from the economic burden consideration, we suggest that the joint utilization of CysC and C1q should be prioritized for the rapid detection of LNA, which warrants further investigations in clinical settings. Nevertheless, due to the aforementioned limitations, our findings need to be confirmed in a larger and more specifically selected LN population in the future.

## Data Availability

The data generated and analyzed will be made available to interested readers.

## Abbreviations

ACR: American College of Rheumatology
ALN: Activity lupus nephritis
AKI: Acute kidney injury
AUC: Area under the curve
ANOVA: One-way analysis of variance
Clq: Component 1q
CysC: Cystatin C
CKD: Chronic kidney disease
eGFR: Estimated glomerular filtration rate
ELISA: Enzyme-linked immunosorbent assay
ESKD: End-stage kidney disease
GFR: Glomerular filtration rate
HC: Healthy controls
ILN: Inactivity lupus nephritis
KIDGO: Kidney Disease Improving Global Outcomes
LN: Lupus nephritis
M: Median
MDRD: Modification of diet in renal disease
ROC: Receiver operating characteristic curve
SD: Standard deviation
SLE: Systemic lupus erythematosus
SLEDAI: SLE disease activity index
UACR: Urinary albumin/creatinine ratio
VDRL: Venereal disease research laboratory test
YI: Youden index
